# Pharmacy delivery to expand the reach of PrEP in Africa

**DOI:** 10.1002/jia2.25619

**Published:** 2020-09-30

**Authors:** Katrina F Ortblad, Peter Mogere, Elizabeth Bukusi, Kenneth Ngure, Jared M Baeten

**Affiliations:** ^1^ Department of Global Health University of Washington Seattle WA USA; ^2^ Partners in Health and Research Development Thika Kenya; ^3^ Centre for Microbiology Research Kenya Medical Research Institute Nairobi Kenya; ^4^ Department of Obstetrics and Gynecology University of Washington Seattle WA USA; ^5^ Department of Community Health Jomo Kenyatta University of Agriculture and Technology Nairobi Kenya; ^6^ Department of Epidemiology University of Washington Seattle WA USA; ^7^ Department of Medicine University of Washington Seattle WA USA

**Keywords:** PrEP, HIV prevention, pharmacy care, Kenya, stakeholders, implementation science

Several African countries have recently integrated pre‐exposure prophylaxis (PrEP) into their national HIV prevention programmes and are in the process of scaling‐up healthcare facility‐based PrEP delivery [1]. To maximize the public health benefit of PrEP, there is need to prioritize access, minimize the costs of delivery and reach HIV at‐risk populations. Major barriers to facility‐based PrEP delivery exist, including facility‐associated HIV stigma, long waiting times, the costs of staffing and providers’ unfamiliarity with delivering prevention interventions [2]. In Africa, PrEP is also being added to public health infrastructures that are sometimes burdened by overcrowding and drug stock outs [3]. Thus, the ability of African health systems to maximize PrEP access necessitates finding novel models of PrEP delivery.

In low‐resource settings, including a number of African settings, private pharmacies fill an important gap in the medical system and individuals often rely on and prefer the use of pharmacies over healthcare facilities to address their medical needs [4]. Pharmacies can address care needs that are both urgent (e.g. evaluation and medication for sexually transmitted infections) and preventive (e.g. contraception) [5] and have advantages over healthcare facilities, including increased convenience and provider engagement. Compared to providers at healthcare facilities, providers at pharmacies can often spend more time with clients because they do not have to focus on treating sick patients and build better rapport with clients because they are for‐profit businesses that rely on repeat services. In low‐resource settings, it is common for individuals to first go to a pharmacy to address a medical issue (e.g. symptoms of malaria), then only go to a healthcare facility later if the issue is not resolved [6, 7, 8].

Delivery of PrEP through pharmacies is one approach being utilized in the US to improve PrEP accessibility. In Seattle, the Kelley‐Ross Pharmacy (a private pharmacy) has developed One Step PrEP, which allows pharmacists to prescribe and manage PrEP care under a collaborative‐practice agreement with a local primary care clinic [9]. Thus far this model has been highly successful; from March 2015 to February 2018, 714 clients were evaluated and 695 (97%) initiated PrEP at the Seattle pharmacy. Among clients that initiated PrEP, 74% received PrEP drugs on the same day of their visit, and among clients that refilled PrEP, 90% were found to be PrEP adherent (i.e. their mean proportion of days covered was >80%). Additionally, no clients HIV seroconverted during the period of pharmacy‐based PrEP delivery. The success of this collaborative practice agreement for pharmacy PrEP care has inspired replication in other US setting (e.g. Omaha, Nebraska and San Francisco, California) to expand PrEP access and continuation [10].

Development of a similar model for pharmacy‐based PrEP delivery in African settings, adapted to local context, could benefit many. Strategic planning for how such a model could be delivered safely and effectively could head off unregulated development of ad hoc PrEP delivery in pharmacies (potentially accompanied with PrEP misinformation). The delivery of PrEP at private pharmacies in Africa is feasible and within the domain of care for pharmacy providers. PrEP delivery has relatively few necessary components – HIV testing, counselling (on PrEP adherence and HIV risk reduction), PrEP prescribing (including assessment of acute HIV infection and PrEP side effects) and drug dispensing (Figure 1) [11] – all of which can be done by pharmacists or pharmaceutical technologists in low‐resource settings (especially with remote clinician oversight, like the US model) [12]. Already, many private pharmacies counsel clients on the importance of adherence to medications for hypertension and diabetes, as well as the importance of condom use for pregnancy and sexually transmitted infection prevention. Some pharmacies in select Africa countries additionally provide access to HIV self‐testing (which can be provider‐assisted) and/or controlled substances (e.g. repeat prescriptions for opioids or epilepsy medications), which require special training, storage and records for dispensing [13].

**Figure 1:**
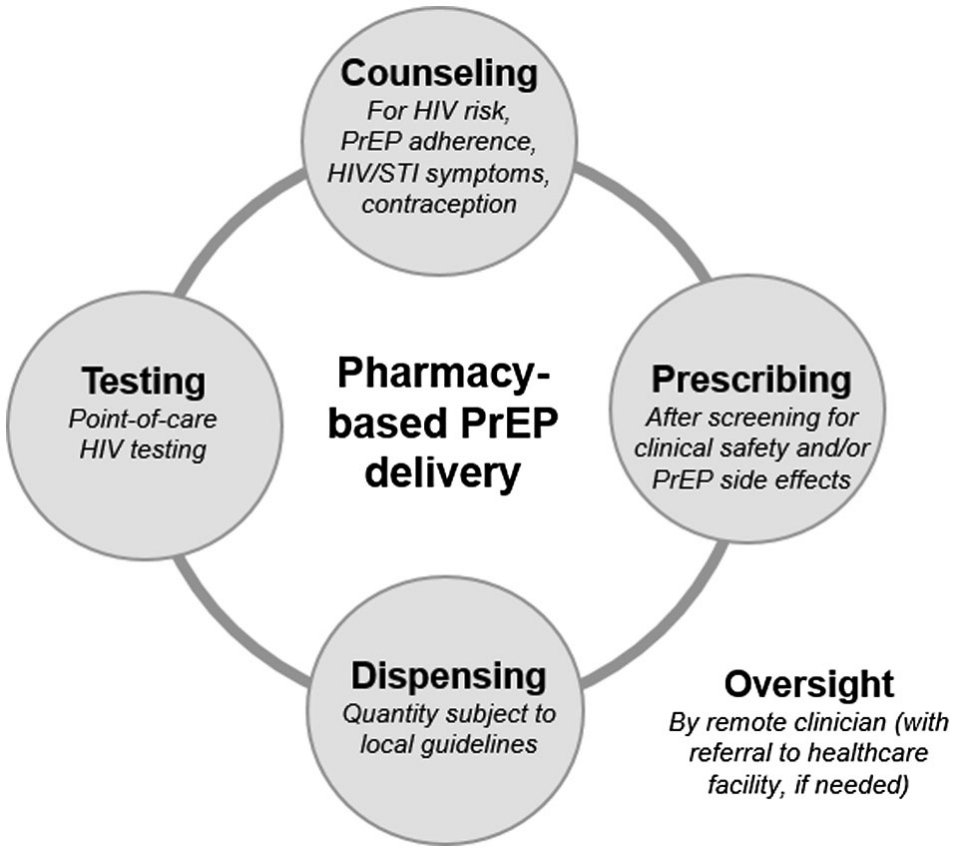
Necessary components of pharmacy‐based PrEP delivery

Compared to the existing facility‐based model of PrEP delivery in Africa, pharmacy‐based PrEP delivery has a number of potential advantages. First, pharmacies outnumber healthcare facilities in any given location and thus might be nearer to individuals interested in PrEP, saving both time and resources. Second, individuals visit pharmacies for both non‐medical and medical reasons, potentially enabling individuals who seek PrEP to maintain privacy and overcome barriers associated with facility‐based PrEP stigma. Third, pharmacies are self‐sustaining by offering subsidized or fee‐for‐service care, which may make them more responsive to client demands and result in sustained client engagement if individuals value services purchased [14]. Finally, the delivery of PrEP in pharmacies expands the choice of locations to access PrEP, enabling individuals to select their preferred model.

Pharmacy‐based PrEP delivery in Africa, however, also presents challenges. First, pharmacy providers in Africa have not been trained on PrEP delivery, and thus will need focused training and potential oversight by a remote clinician. Second, a necessary cost will be associated with PrEP access at pharmacies to make the model self‐sustaining, which may exclude some individuals (but may also engage other individuals who value services purchased [14]). Third, individuals may forgo screening for treatment of other health conditions (e.g. hypertension, diabetes) if they are no longer frequenting healthcare facilities for PrEP services. Some of these screening services, however, could be moved to pharmacy settings and paired with linkage to care interventions for clients identified as at risk for a particular health condition. Finally, maintaining clients’ privacy at smaller pharmacies with limited space may be a challenge. Thus, only pharmacies with adequate space and the availability of a private room for client counselling and HIV testing should be allowed to deliver PrEP services.

To end the HIV epidemic, access to HIV prevention and treatment services must increase in high HIV prevalence African settings. Standard facility‐based models of HIV prevention and care are not currently reaching all the populations at risk of or living with HIV. The global coronavirus disease 2019 (COVID‐19) epidemic further pushes health systems to consider more client‐friendly services. Pharmacy‐based PrEP delivery in Africa is timely and has great potential to reach individuals not currently engaged in PrEP care and make PrEP more accessible to individuals at risk of HIV infection.

## COMPETING INTEREST

The authors of this Viewpoint have no conflicts of interest to declare.

## AUTHORS’ CONTRIBUTIONS

All authors developed the points in the Viewpoint together over multiple discussions. K.F.O. drafted the Viewpoint. All authors reviewed and edited the final Viewpoint.
